# LncRNA LEF1-AS1 exerts a carcinogenic effect in breast cancer by accelerating proliferation, metastasis, and epithelial-mesenchymal transition

**DOI:** 10.1186/s41065-025-00613-2

**Published:** 2025-11-29

**Authors:** Linna Kong, Jiaqi Liu, Huihui Zhang, Jun Chu

**Affiliations:** Department of Breast and Thyroid Surgery, Zibo Central Hospital, 54 Gongqingtuan West Road, Zibo, Shandong 255020 China

**Keywords:** Breast cancer, LncRNA, LEF1-AS1, Cancer metastasis, Malignant proliferation, EMT, Competing endogenous RNA

## Abstract

**Background:**

Breast cancer (BRCA) directly poses a threat to human life safety. This study aims to explore the role of lncRNA LEF1-AS1 in BRCA.

**Methods:**

Firstly, the levels of LEF1-AS1 in tumor tissues and BRCA cells were detected by qRT-PCR. Furthermore, LEF1-AS1 was inhibited through cell transfection. Subsequently, the proliferation, apoptosis and migration abilities of the cells were detected using the CCK-8 assay, flow cytometry and Transwell assay. Meanwhile, the mRNA levels of epithelial-mesenchymal transition (EMT) markers were also measured. The targeting binding relationships between LEF1-AS1 and miR-328-5p, as well as between miR-328-5p and KLF16, were verified by dual luciferase reporter assays and RNA pull-down assays. Furthermore, the effects of co-regulating LEF1-AS1 and miR-328-5p and down-regulating KLF16 on tumor cells were further investigated.

**Results:**

In tumor tissues and BRCA cell lines, LEF1-AS1 is highly expressed. Downregulation of LEF1-AS1 inhibits the malignant proliferation of BRCA cells and reduces their migration ability. Moreover, the process of EMT is significantly inhibited. miR-328-5p is the downstream target of LEF1-AS1. Simultaneously inhibiting miR-328-5p and LEF1-AS1 weakens the anti-cancer effect resulting from LEF1-AS1 silencing. LEF1-AS1 mainly participates in tumor progression by acting as a sponge molecule for miR-328-5p. Inhibiting LEF1-AS1 leads to the downregulation of KLF16, while the inhibition of miR-328-5p leads to the upregulation of KLF16. Moreover, the downregulation of KLF16 will result in weakened proliferation and migration abilities of BRCA cells.

**Conclusions:**

LEF1-AS1 increases the level of KLF16 by sponging miR-328-5p, thereby promoting the proliferation, metastasis and EMT of BRCA cells.

**Supplementary Information:**

The online version contains supplementary material available at 10.1186/s41065-025-00613-2.

## Background

Breast cancer (BRCA) is a malignant tumor that originates from the epithelial cells of the breast [1]. The Global Cancer Statistics Report 2025 shows that BRCA has ranked first in the incidence rate of female malignant tumors for six consecutive years. Although the incidence rate of BRCA among men is only 1%, due to carelessness, this situation often leads to delays in diagnosis and treatment. In advanced stages of BRCA, nipple bleeding, severe breast pain, and other symptoms may occur [2]. Additionally, bone metastasis, lung metastasis, liver metastasis, and distant metastasis caused by BRCA significantly reduce the survival rate of patients [3].

Apart from directly endangering one’s physical health, BRCA also causes significant psychological trauma to patients and imposes a heavy financial burden on their families. However, an encouraging aspect is that with the breakthroughs in molecular biology and imaging technologies, the diagnostic accuracy of BRCA has significantly improved. For instance, the detection rate of digital breast tomography synthesis technology has been significantly higher than that of traditional detection methods [4]. At the level of liquid biopsy, markers such as circulating tumor DNA (ctDNA) and exosomes have also demonstrated extremely high sensitivity in the diagnosis of BRCA [5, 6]. Given the numerous adverse consequences caused by BRCA, in-depth exploration of the pathogenesis of BRCA is of great significance for improving treatment outcomes, developing new therapies, and achieving personalized medicine and precise prevention.

With the advancement of the ENCODE project, it has been confirmed that 98% of the non-coding regions in the human genome have regulatory functions. These non-coding RNAs are not only used for the development of diagnostic markers, but also for the formulation of targeted treatment strategies [7, 8]. Non-coding RNAs have been discovered to participate in gene expression regulation through various mechanisms such as epigenetic regulation [9], chromatin remodeling [10], and competitive endogenous RNA mechanisms [11], and are closely related to the pathogenesis of tumors [12], neurodegenerative disorders [13], and cardiovascular diseases [14].

In BRCA, lncRNA play a crucial regulatory role in the development and prognosis of the disease. The mechanism involves multiple aspects such as cell proliferation, metastasis, and remodeling of the immune microenvironment [15]. For instance, lncRNA MALAT1 has been proven to be a key regulatory factor in BRCA. This lncRNA is widely expressed in BRCA cells and affects the progression and treatment outcome of BRCA by influencing angiogenesis, as well as the invasiveness and drug resistance of cancer cells [16]. In another study, lncRNA XIST increases the expression of epithelial-mesenchymal transition (EMT) markers in BRCA [17]. It is noteworthy that EMT plays a key role in BRCA. The occurrence of EMT can enable cancer cells to acquire migration and invasion abilities by upregulating the expression of mesenchymal markers [18]. In previous studies, lncRNA LEF1-AS1 was proven to be related to various cancers including colorectal cancer and prostate cancer and involved multiple regulatory mechanisms [19, 20]. However, the role of this molecule in BRCA still needs further exploration.

In this study, we investigated the role of lncRNA LEF1-AS1 in BRCA from the perspectives of proliferation, migration and EMT. The aim was to deepen the understanding of the mechanism of BRCA and to develop new targets for the diagnosis and treatment of BRCA.

## Methods

### Breast tumor sample

From June 2021 to June 2023, this study collected tumor tissues from 90 breast cancer patients treated at Zibo Central Hospital. At the same time, normal breast tissues 5 cm away from the tumor tissues were also collected. Before the analysis began, all the tissues were stored in a −80℃ ultra-low temperature refrigerator. This study has been approved by the Zibo Central Hospital Ethics Committee. All patients provided informed consent forms.

### Breast cancer cell line

Human breast epithelial cell line (MCF-10 A) and the human breast cancer cell lines (MDA-MB-231, MCF-7, T-47D) were purchased from the Chinese Academy of Sciences Cell Bank (China). The MCF-10 A, MDA-MB-231 and MCF-7 cells were cultured in DMEM medium (Gibco, USA) containing 10% fetal bovine serum (FBS) and 1% penicillin-streptomycin double antiserum (Invitrogen, USA). The T-47D cells were grown in RPMI-1640 medium (Gibco, USA). The culture conditions for all cells are 37℃ and 5% CO2. 

### qRT‑PCR

By using the TRIzol reagent (Invitrogen, USA), we first extracted total RNA from the collected cells. Subsequently, we used PrimeScript RT reagent (TAKARA, Japan) to perform reverse transcription. Finally, we prepared the qPCR reaction solution containing cDNA, TB Green and primers to specifically amplify the target gene. β-actin and U6 were the internal reference genes in the quantitative process of the target gene. The information of the primers is provided in the Table S1.

### Cell transfection

According to the different experimental requirements, inoculate the appropriate number of cells onto the cell culture plates. When the cells are in the logarithmic growth phase, prepare the transfection reagents, plasmid vectors, miRNA inhibitors and miRNA mimics dilutions and suspensions according to the instructions for using Lipofectamine 3000 (Invitrogen, USA). Then, incubate the suspension with the cells together. After 48 h, collect the cells.

### CCK-8 assay

Firstly, the cells were inoculated into 96-well plates and cultured under suitable conditions for 0 h, 24 h, 48 h, and 72 h. Subsequently, 10 µL of the CCK-8 solution (Beyotime, China) was added to each well. After incubation at 37 °C for 2 h, the detection wavelength of the microplate reader (BioTek, USA) was set to 450 nm, and the absorbance value of the samples was measured.

### Measurement of apoptosis rate

First, the cells should be washed with PBS. Then, resuspend the cells in 1×Binding Buffer and add Annexin V-FITC and PI Staining Solution. After mixing, incubate the sample in the dark for 15 min. Finally, add 1× Binding Buffer and immediately perform apoptosis cell detection using a flow cytometer. All reagents are included in the Annexin V-FITC/PI Apoptosis Detection kit (YEASEN, China).

### Transwell assay

12 h before the experiment, the cell culture medium was replaced with serum-free medium to put the cells in a starvation state. Then, 200 µL of cell suspension was inoculated into the upper chamber of the Transwell chamber (Corning, USA). 600 µL of complete medium containing fetal bovine serum was added to the lower chamber of the Transwell chamber. After culturing in the incubator for 24 h, the Transwell chamber was taken out, and the culture medium was removed and the cells that did not migrate were removed with a cotton swab. Then, the Transwell chamber was fixed with 4% paraformaldehyde for 20 min. The chamber was washed twice with PBS and then placed in 0.1% crystal violet solution for staining for 30 min. After the staining was completed, it was washed twice with PBS. Finally, 5 fields of view were randomly selected under the microscope and the number of cell migrations was observed.

### Subcellular fractionation

The procedure for separating the nucleus and cytoplasm of BRCA cells was carried out exactly in accordance with the instructions provided in the PARIS™ Kit (Life Technologies, USA). The specific operation steps are as follows: Add Cell Fractionation Buffer to the BRCA cells and incubate them on ice for 10 min. Then, centrifuge at 500xg and 4℃ for 5 min. At this point, the liquid in the centrifuge tube is divided into two parts. The bottom sediment is the cell nucleus, and the supernatant is the lysate containing the cytoplasm. To obtain the lysate of the cell nucleus, add an appropriate amount of Cell Disruption Buffer to the sediment. Then, add 2X Lysis/Binding Solution to the centrifuge tubes. Then, the sample is filtered through the filter cylinder and the RNA is eluted using the Elution Solution. Finally, determine the RNA in the cell nucleus and cytoplasm separately by qRT-PCR.

### RNA pull-down assay

To verify whether lncRNA LEF1-AS1 can bind to miR-328-5p, we conducted a miRNA pull-down experiment. Firstly, BersinBio (China) was commissioned to synthesize biotin-labeled miRNA probes. The miRNA probes were designed based on the sequence of miR-328-5p, and they were consistent with the mature sequence of miR-328-5p and were labeled with biotin. These probes can bind to streptavidin magnetic beads under in vitro conditions. After obtaining the probes, these miRNA probes were transfected into cells. After 48 h, the cells were lysed and the cell lysate was mixed with streptavidin magnetic beads to obtain the magnetic bead-miRNA-RNA complex. Finally, the target complex was eluted using a magnetic rack and the enrichment level of LEF1-AS1 in the complex was determined by qPCR. Control experiments were carried out using a scrambled miRNA probe to validate assay specificity.

### Dual-Luciferase Reporter Assay

The LEF1-AS1 and KLF16 sequences containing the binding sites or mutation sites of miR-328-5p were inserted into the pmirGLO plasmid (Promega, USA) to construct a dual luciferase reporter plasmid (LEF1-AS1-WT, LEF1-AS1-MUT, KLF16-WT, KLF16-MUT). When the cell confluence reached 60%, the reporter plasmid and miRNA mimics were transfected into the cells using Lipofectamine 3000. The Dual Luciferase Assay System (Promega, USA) was used to detect the luciferase activity.

### Bioinformatics analysis

By utilizing the GEPIA database and the UALCAN database, which are two public databases related to cancer, this study analyzed the expression levels of LEF1-AS1 in breast cancer tissues and normal breast tissues. Additionally, using the LncRNASNP database and the miRDB database respectively, this study predicted the downstream targets of LEF1-AS1 and miR-328-5p.

### Statistical analysis

All statistical analyses were performed using GraphPad Prism 9.0. The threshold for statistical significance was set at *p* < 0.05 (two-tailed). Data are presented as mean ± standard deviation (SD) or number (percentage). Continuous variables were analyzed using the independent samples t-test. The comparison of categorical variables is conducted using the χ² test. Correlation was analyzed by Pearson correlation analysis. Confounding factors were controlled by linear regression analysis.

## Results

### The clinical application value of LncRNA LEF1-AS1 in BRCA

**Fig. 1:**
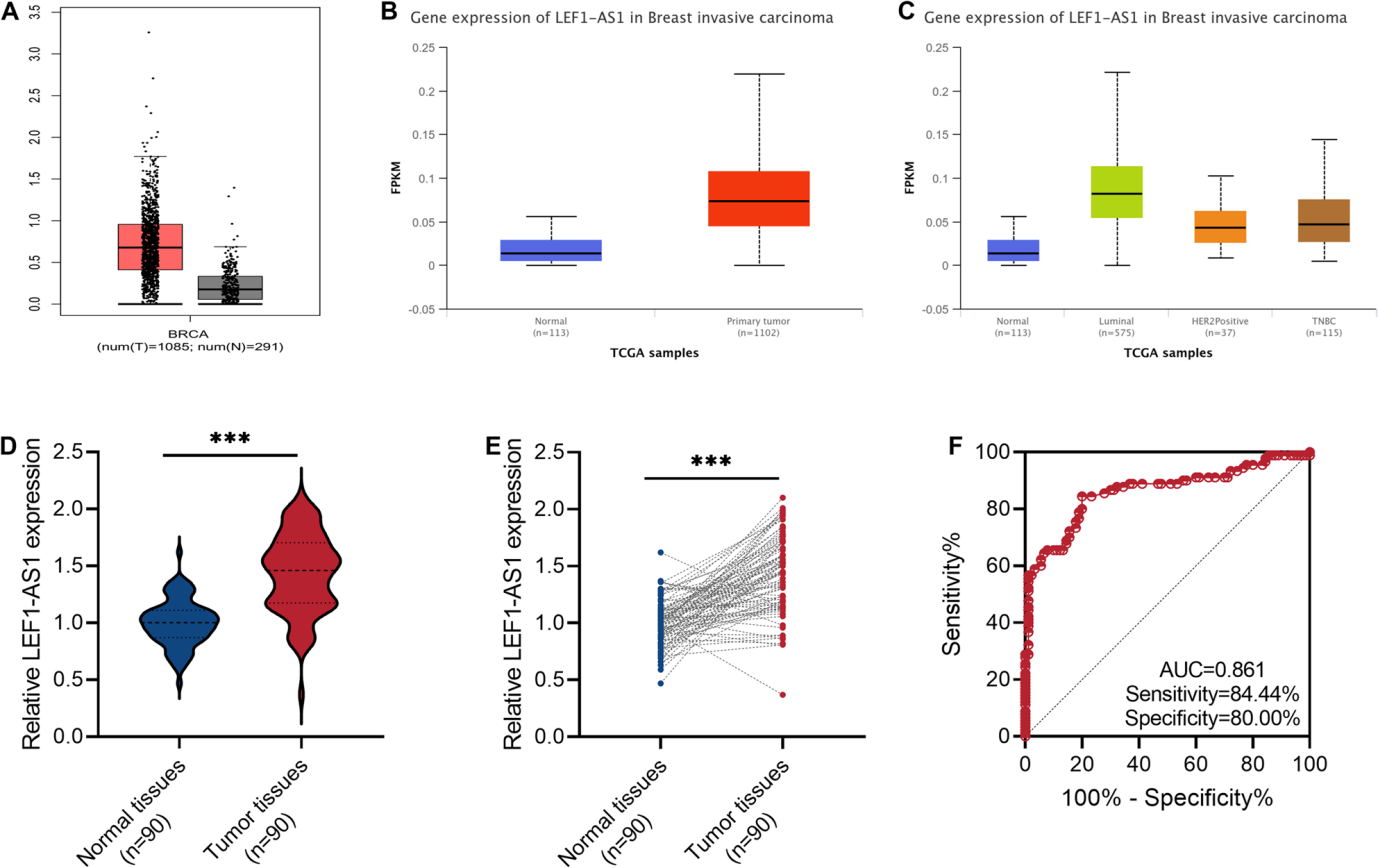
The lncRNA LEF1-AS1 is persistently highly expressed in the tumors of breast cancer (BRCA) patients. **A** The GEPIA database shows the expression trend of LEF1-AS1 in the context of BRCA. **B** Expression status of LEF1-AS1 in unpaired samples from TCGA-BRCA dataset. **C** The expression status of LEF1-AS1 in different molecular subtypes of BRCA samples recorded in the UALCAN database. **D**-**E**. The expression levels of LEF1-AS1 in paired BRCA tissues and normal tissues were detected by qRT-PCR. **F**. The ability of LEF1-AS1 to diagnose and differentiate BRCA was analyzed by drawing the Receiver Operating Characteristic (ROC) curve. ***P < 0.001

To initially explore the role of lncRNA LEF1-AS1 in BRCA, we first analyzed the expression level of this molecule by referring to the GEPIA and UALCAN databases. As shown in Fig. 1A and B, lncRNA LEF1-AS1 was highly expressed in BRCA samples. Moreover, in different molecular subtypes, this molecule showed an increasing trend (Fig. 1C). Subsequently, we collected 90 tumor samples from clinical cases and re-analyzed the expression level of this molecule. Similarly, LEF1-AS1 was continuously highly expressed in tumor tissues (*P* < 0.001, Fig. 1D and E). Additionally, LEF1-AS1 has a high diagnostic value for breast tumors. The AUC value of LEF1-AS1 for diagnosing BRCA was 0.861, with a specificity of 80.00% and a sensitivity of 84.44% (cut-off value = 1.125, Fig. 1F). Based on the average expression level of LEF1-AS1 (Average value = 1.43), 90 BRCA patients were divided into a high-expression group (≥ 1.43) and a low-expression group (<1.43). This study also found that higher levels of LEF1-AS1 were closely related to larger tumors and higher lymph node metastasis (*P* < 0.05, Table 1).

**Table 1:** Correlation between LEF1-AS1 expression and the clinicopathological features of breast cancer

Features	LEF1-AS1 expression		*P*-value
	Low (*n* = 40)	High (*n* = 50)	
Age			0.397
< 50	18	18	
≥ 50	22	32	
Tumor size (cm)			
≤ 2	22	16	0.034
> 2	18	34	
Lymph node metastasis			0.036
Negative	26	21	
Positive	14	29	
TNM stage			0.073
I-II	35	35	
III-IV	5	15	
ER expression			0.830
Negative	16	22	
Positive	24	28	
PR expression			0.144
Negative	16	28	
Positive	24	22	
HER-2 expression			0.512
Negative	23	33	
Positive	17	17	

*P < 0.05

### Down-regulation of LEF1-AS1 has a strong inhibitory effect on BRCA cells

**Fig. 2:**
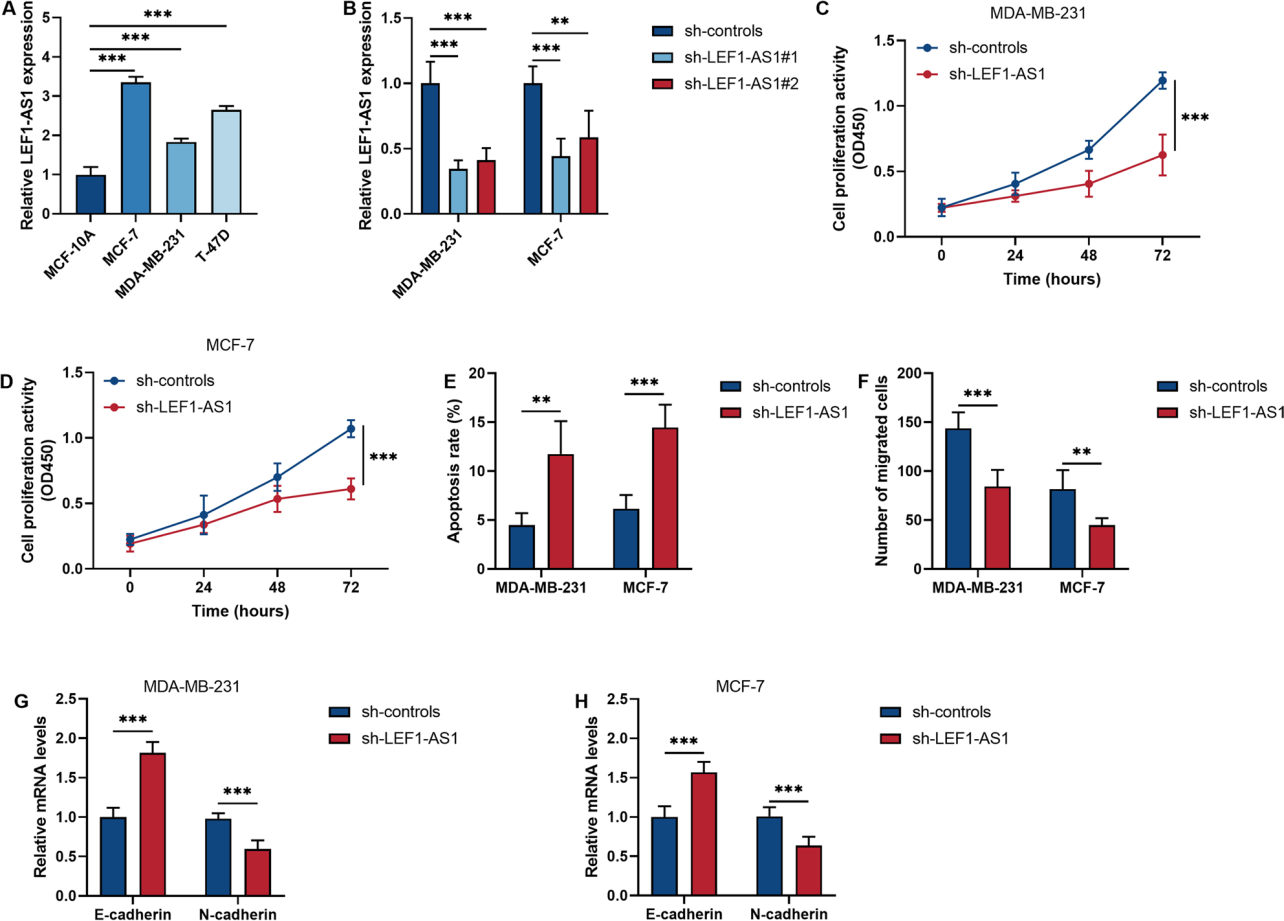
Down-regulation of LEF1-AS1 can inhibit the malignant biological behavior of breast cancer (BRCA) cells. **A** The level of LEF1-AS1 in normal breast epithelial cells and BRCA cells. **B** The inhibitory efficiency of sh-RNA on LEF1-AS1 was detected by qRT-PCR. **C**-**D**. The effect of down-regulating LEF1-AS1 on the proliferation of BRCA cells was detected by CCK-8 assay. **E**. The apoptotic level of cells was determined by flow cytometry. **F**. The effect of inhibiting LEF1-AS1 on cell mobility was investigated through the Transwell assay. **G**-**H**. The expression levels of epithelial-mesenchymal transition markers in two BRCA cell lines. *P < 0.05. **P < 0.01. ***P < 0.001

The lncRNA LEF1-AS1 is also highly expressed in the BRCA cell line (*P* < 0.001, Fig. 2A). Subsequently, in order to verify the impact of lncRNA LEF1-AS1 on the biological behavior of BRCA cells, we conducted a functional knockout experiment. The sh-RNA significantly reduced the level of LEF1-AS1 in BRCA cells (*P* < 0.01, Fig. 2B). This study found that after down-regulating LEF1-AS1, the proliferation ability of BRCA cells significantly decreased while the apoptosis rate increased (*P* < 0.01, Fig. 2C and E). Moreover, down-regulation of LEF1-AS1 significantly inhibited cell migration and epithelial-mesenchymal transition (*P* < 0.01, Fig. 2F and H).

### LEF1-AS1 May act as a sponge for miR-328-5p

**Fig. 3:**
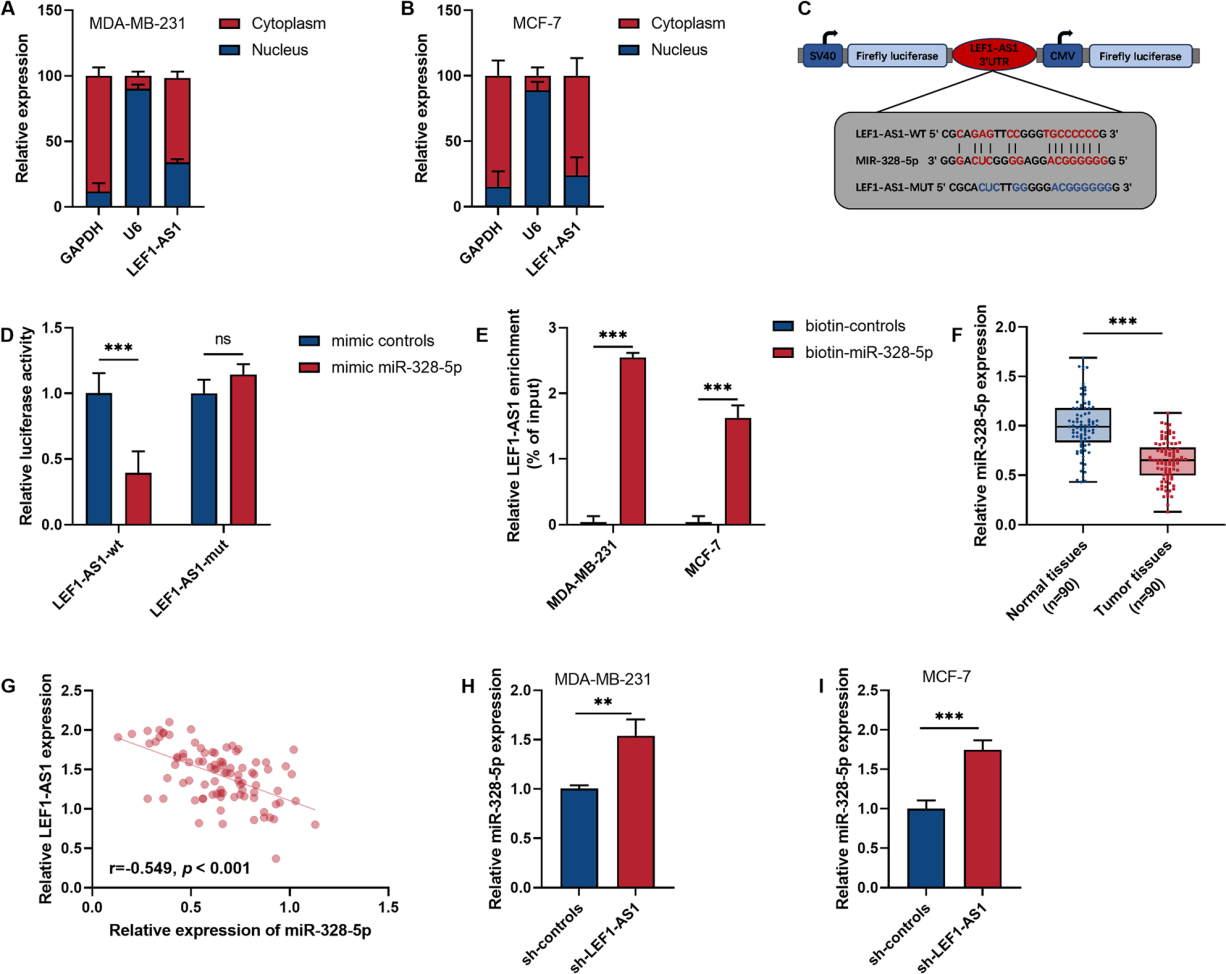
LEF1-AS1 targets and binds to miR-328-5p. **A**-**B**. The localization of LEF1-AS1 in breast cancer (BRCA) cell lines was verified through Subcellular fractionation. **C**. The binding site of miR-328-5p in the LEF1-AS1 sequence. **D**. Dual-luciferase reporter assay. **E**. RNA pull-down assay. **F**. The expression levels of miR-328-5p in paired BRCA tissues and normal tissues were detected by qRT-PCR. **G**. The correlation between miR-328-5p levels and LEF1-AS1 levels in tumor tissues. **H**-**I**. The effect of reducing LEF1-AS1 on the expression of miR-328-5p. *P < 0.05. **P < 0.01. ***P < 0.001

To preliminarily infer the regulatory mechanism of LEF1-AS1, we analyzed the distribution of this molecule in BRCA cells. This study confirmed that LEF1-AS1 is mainly expressed in the cytoplasm (Fig. 3A and B). Therefore, we hypothesized that this molecule may mainly exert its function by acting as a sponge for miRNA. As shown in Fig. 3C, LEF1-AS1 can specifically bind to miR-328-5p. Experimental results demonstrated that transfection of miR-328-5p mimics significantly inhibited the transcription of luciferase in LEF1-AS1-wt (*P* < 0.001, Fig. 3D). Furthermore, the biotin-labeled miR-328-5p was able to significantly enrich LEF1-AS1 (*P* < 0.001, Fig. 3E). The level of miR-328-5p in BRCA tumors was significantly reduced (*P* < 0.001, Fig. 3F). Furthermore, the Pearson correlation analysis revealed a significant negative correlation between miR-328-5p and LEF1-AS1 (*r*=−0.549, *P* < 0.001, Fig. 3G). After controlling for confounding factors using linear regression analysis, the correlation between miR-328-5p and LEF1-AS1 still existed (β=−0.523, *P* < 0.001, Table S2). In cells, this study confirmed that reducing the level of LEF1-AS1 could lead to an increase in miR-328-5p (*P* < 0.01, Fig. 3H and I).

### Inhibition of miR-328-5p weakened the inhibitory effect of Silencing LEF1-AS1 on BRCA cells

**Fig. 4:**
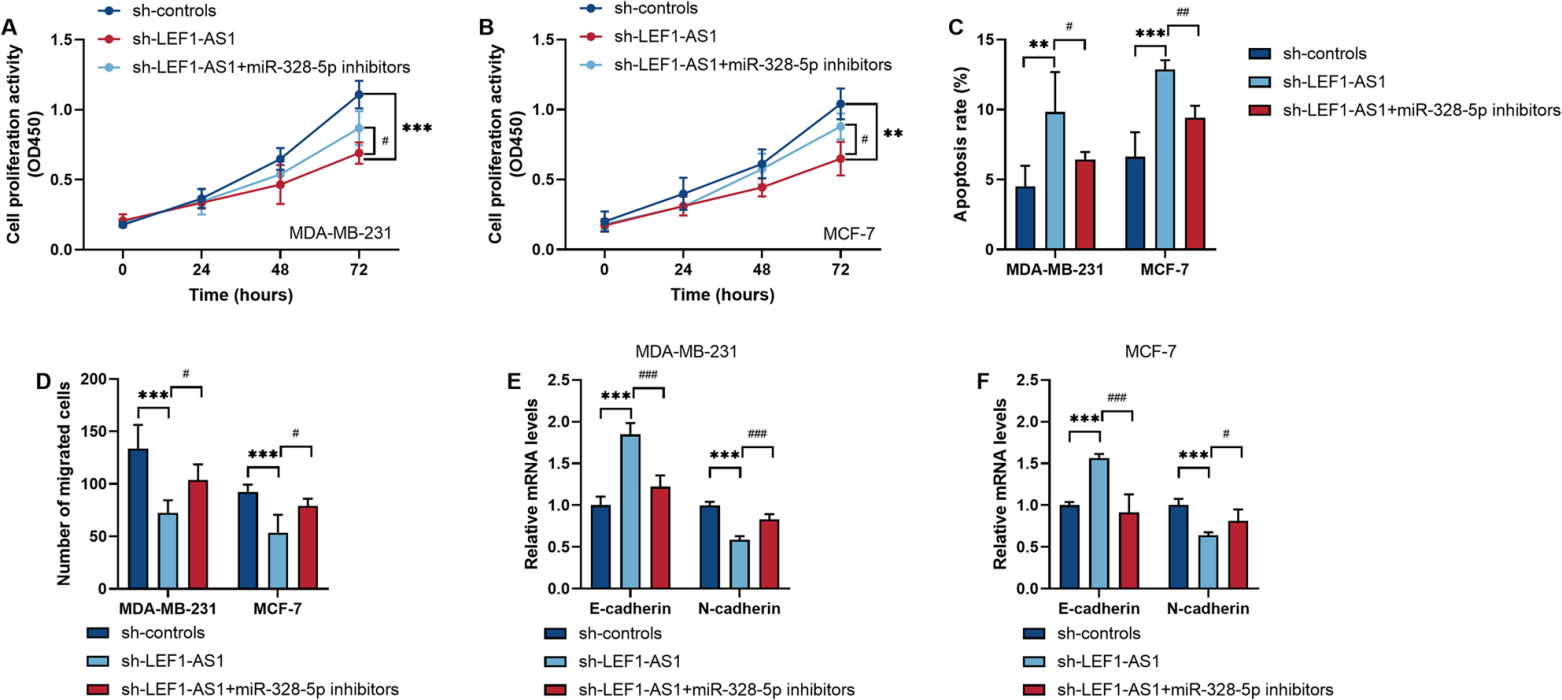
Simultaneously inhibiting LEF1-AS1 and miR-328-5p in breast cancer (BRCA) cell lines weakened the anti-cancer effect caused by the downregulation of LEF1-AS1. **A**-**B**. Measurement of cell proliferation activity. **C**. Measurement of apoptosis rate. **D**. Measurement of cell migration ability. **E**-**F**. The expression levels of epithelial-mesenchymal transition markers in two BRCA cell lines. *P < 0.05. **P < 0.01. ***P < 0.001. ^#^*P* < 0.05. ^##^*P* < 0.01. ^###^*P* < 0.001

By simultaneously inhibiting the expression of LEF1-AS1 and miR-328-5p in BRCA cells, we verified whether LEF1-AS1 participates in regulating the malignant biological behaviors of cancer cells by targeting miR-328-5p. In BRCA cells, reducing the expression of LEF1-AS1 can inhibit cell proliferation and increase the apoptosis rate (*P* < 0.01, Fig. 4A and C). However, down-regulating miR-328-5p on this basis instead up-regulated cell proliferation and led to a decrease in the apoptosis rate (*P* < 0.05, Fig. 4A and C). During the processes of cell migration and Epithelial-mesenchymal transition (EMT), this study found that simultaneously inhibiting LEF1-AS1 and miR-328-5p weakened the reduction in migration rate and the weakening of EMT caused by the decrease in LEF1-AS1 level (*P* < 0.05, Fig. 4D and F).

### LEF1-AS1 enhances the expression of KLF16 by sponging miR-328-5p, thereby promoting the progression of BRCA

**Fig. 5:**
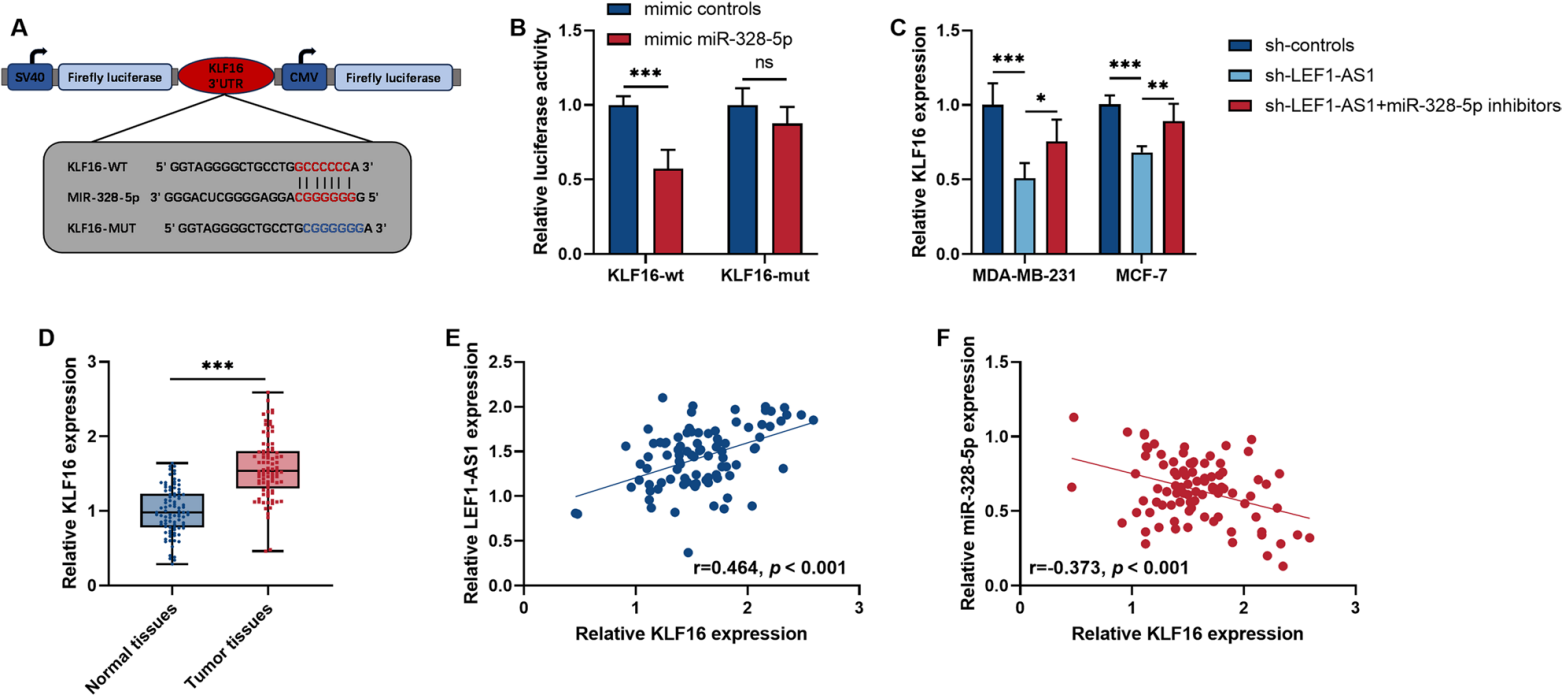
LEF1-AS1 indirectly regulates KLF16 by sponging miR-328-5p. **A** The binding site of miR-328-5p in the KLF-16 sequence. **B** Dual-luciferase reporter assay. **C** The influence of regulating LEF1-AS1 and miR-328-5p in BRCA cell lines on the level of KLF16. **D** The expression levels of KLF16 in paired BRCA tissues and normal tissues were detected by qRT-PCR. **E** The correlation between KLF16 levels and LEF1-AS1 levels in tumor tissues. **F** The correlation between KLF16 levels and miR-328-5p levels in tumor tissues. *P < 0.05. **P < 0.01. ***P < 0.001

In further studies, we identified KLF16 as the downstream target of miR-328-5p by using the miRDB database (Fig. 5A). Transfection of miR-328-5p mimics in cells significantly inhibited the luciferase activity of KLF16-wt (*P* < 0.001, Fig. 5B). This proved that miR-328-5p can indeed bind to the sequence of KLF16. Additionally, we confirmed that downregulation of LEF1-AS1 leads to a decrease in KLF16, and inhibition of miR-328-5p expression can partially restore the level of KLF16 (*P* < 0.05, Fig. 5C). Clinical data also confirmed that KLF16 is increased in tumors (*P* < 0.001, Fig. 5D). Moreover, KLF16 shows a positive correlation with LEF1-AS1 (*r* = 0.464, *P* < 0.001, Fig. 5E) and a negative correlation with miR-328-5p (*r*=−0.373, *P* < 0.001, Fig. 5F). After controlling for confounding factors through linear regression analysis, the correlations between KLF16 and miR-328-5p (β = −0.188, *P* < 0.05, Table S2), as well as between KLF16 and LEF1-AS1 (β = 0.380, *P* < 0.01, Table S2), remained significant. Therefore, LEF1-AS1 is highly likely to upregulate KLF16 by sponging miR-328-5p, and ultimately promote the cancer phenotype.

**Fig. 6:**
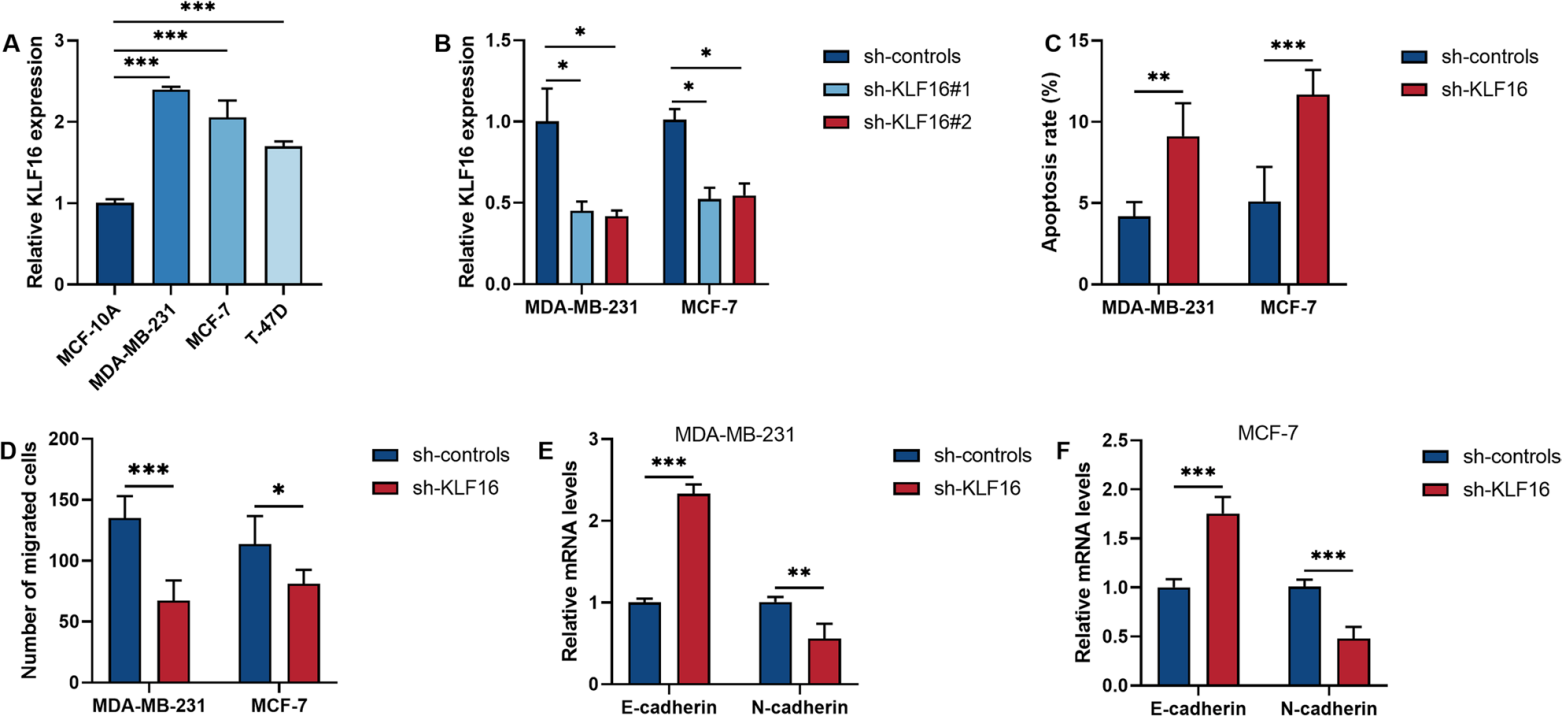
KLF16 is highly expressed in breast cancer (BRCA) cell lines. **A** The level of KLF16 in normal breast epithelial cells and BRCA cells. **B** The inhibitory efficiency of sh-RNA on KLF16 was detected by qRT-PCR. **C** Measurement of apoptosis rate. **D** Measurement of cell migration ability. **E**-**F**. The expression levels of epithelial-mesenchymal transition markers in two BRCA cell lines. *P < 0.05. **P < 0.01. ***P < 0.001

Finally, we verified the impact of changes in KLF16 levels on cell functions in BRCA cells. The BRCA cell lines all expressed high levels of KLF16 (*P* < 0.001, Fig. 6A). Moreover, inhibiting the overexpression of KLF16 in BRCA cells significantly promoted cell apoptosis while inhibiting cell migration and the EMT process (*P* < 0.05, Fig. 6B and F).

## Discussion

LncRNA have been proven to be abnormally expressed in various tumor tissues and can serve as specific diagnostic markers for cancer. For instance, the identification of MALAT1 in tumor tissues of lung cancer, ovarian cancer and prostate cancer can be used as an auxiliary diagnostic method [21]. LncRNA can also stably exist in body fluids such as blood and urine and be transmitted through exosomes [22, 23]. Therefore, the combination of specific lncRNA in blood can also be used as a non-invasive method for early cancer screening. Besides, lncRNA are often used as an indicator for prognosis assessment. For instance, in triple-negative breast cancer, DDIT4-AS1 and H19 are significantly associated with tumor metastasis, recurrence and chemotherapy resistance [24, 25]. As a tumor suppressor, LINC00261 is closely related to the clinical characteristics of patients and represents a potential therapeutic target [26].

Published studies have shown that in hypopharyngeal squamous cell carcinoma, esophageal squamous cell carcinoma and colorectal cancer, LEF1-AS1, as a metastasis marker, indicates a poor prognosis [27–29]. Patients with high expression of LEF1-AS1 have significantly shorter overall survival and disease-free survival. We speculate that LEF1-AS1 may serve as a promising biomarker in multiple human cancers to indicate prognosis. Our study confirmed that lncRNA LEF1-AS1 shows good diagnostic performance in differentiating BRCA tissues from normal breast tissues. Furthermore, the high level of LEF1-AS1 is significantly correlated with the advanced tumor stage and high metastasis rate in BRCA patients. Thus, our results further enrich and expand this conclusion and fill the gap in the diagnostic application of LEF1-AS1 in BRCA. As a biomarker, LEF1-AS1 can not only be used for the diagnosis of BRCA but also is expected to be used for risk stratification of BRCA.

Cell proliferation and metastasis are the core mechanisms driving cancer progression, and they are interrelated and jointly lead to disease deterioration and increased treatment difficulty. Proliferation is the foundation of early cancer progression. Cancer cells acquire the ability to divide infinitely through gene mutations and are not constrained by normal regulatory mechanisms [30]. On this basis, cancer cells form primary tumors and continue to grow. The formation of metastasis marks the advanced stage of cancer. Cancer metastasis promotes the development of cancer from a local lesion to a systemic disease, which significantly shortens the survival period of patients [31, 32]. Studies have shown that in ovarian cancer and lung cancer, LEF1-AS1 has the ability to promote the proliferation and metastasis of cancer cells [33, 34]. However, in different types of cancer, the same gene may exert different effects. Therefore, we further investigated the role of LEF1-AS1 in the proliferation and metastasis of BRCA cells. We found that in vitro cultured BRCA cell lines, reducing the expression of LEF1-AS1 can significantly inhibit the proliferation rate of cells and curb the migration rate of cells. Therefore, lncRNA LEF1-AS1 may promote the formation or deterioration of BRCA by promoting cancer cell proliferation and activating the metastasis of cancer cells. In BRCA, LEF1-AS1 also exerts a carcinogenic effect.

Regarding the metastasis of cancer cells, EMT plays a role in promoting the detachment of cancer cells from the primary site and their migration. EMT down-regulates the expression of adhesion molecules such as E-cadherin, thereby disrupting cell-cell connections and enabling cancer cells to acquire the ability to migrate [35]. Cancer cells that have undergone EMT can exhibit mesenchymal cell morphology, which is conducive to local infiltration and vascular invasion [36]. Some recent reviews have indicated that certain compounds extracted from plants, such as baicalin and curcumin, have a very significant regulatory effect on N-cadherin and E-cadherin. Moreover, these components have a remarkable anti-cancer effect [37, 38]. Thus, it can be seen that inhibiting the EMT of cancer cells is a crucial step in cancer treatment. Therefore, in addition to directly observing the impact of lncRNA LEF1-AS1 on the biological behavior of BRCA cells, we also studied the effects of lncRNA LEF1-AS1 on E-cadherin and N-cadherin, which are the EMT markers. We found that LEF1-AS1 has the effect of reducing the level of E-cadherin. This is consistent with the result that lncRNA LEF1-AS1 promotes the migration of cancer cells.

We also analyzed the distribution of lncRNA LEF1-AS1 in BRCA cell lines. lncRNA LEF1-AS1 mainly exists in the cytoplasm of BRCA cells. Numerous studies have confirmed that in the cytoplasm, lncRNAs exert their biological functions through the competitive endogenous RNA mechanism [39]. LncRNA contain miRNA response elements (MREs) similar to mRNAs, and can competitively adsorb miRNAs through base complementary pairing, blocking the binding of miRNAs to target gene mRNAs, thereby relieving the inhibitory effect of miRNAs on target genes. When lncRNA expression is activated, it can bind to more miRNAs, upregulating the expression of target genes; when silenced, it leads to the inhibition of target genes [40]. In this study, we confirmed that lncRNA LEF1-AS1 upregulates KLF16 by acting as a molecular sponge for miR-328-5p. KLF16, as a carcinogenic factor, has been confirmed in various cancer-related studies. The high expression of KLF16 continuously promotes the proliferation and invasion of cancer cells and induces angiogenesis [41, 42]. Especially in BRCA, a recent published study has confirmed that high levels of KLF16 are associated with poor prognosis in BRCA patients. Knockout of KLF16 in vivo and in vitro inhibited tumor proliferation [43]. Our research results are in line with this study. We demonstrated that inhibiting KLF16 in BRCA cells can not only promote cell apoptosis but also inhibit cell migration and EMT.

## Conclusions

In summary, the exploration of the role of lncRNA in BRCA is of great significance for revealing the mechanism of tumor progression and developing therapeutic targets. This study confirmed that lncRNA LEF1-AS1 sponges miR-328-5p, thereby relieving the inhibitory effect of miR-328-5p on KLF16. This will be beneficial for the proliferation and distant metastasis of cancer cells.

## Supplementary Information

Supplementary Material 1.

Supplementary Material 2.

## Data Availability

All data generated or analyzed during this study are included in this article. Further enquiries can be directed to the corresponding author.

## Abbreviations

BRCA: Breast cancer
EMT: Epithelial-mesenchymal transition
ctDNA: circulating tumor DNA
lncRNA: long non-coding RNAs
SD: Standard deviation
