# Supplementary material for: LncRNA LEF1-AS1 exerts a carcinogenic effect in breast cancer by accelerating proliferation, metastasis, and epithelial-mesenchymal transition
Source: Hereditas. 2025 Nov 29;163:7. doi: 10.1186/s41065-025-00613-2 (PMC12771952; doi:10.1186/s41065-025-00613-2)
Supplement: Supplementary file 1 — Supplementary Material 1. [file 41065_2025_613_MOESM1_ESM.docx]

Supplementary Table S1. Primers for fluorescence quantitative PCR

| Gene name | Primer information (5’-3’) |
| --- | --- |
| LEF1-AS1 | F: AAGGACGAGAGAAAAGCAC |
|  | R: CACACAAAGGGGAAGACC |
| MiR-328-5p | F: GGGGGGCAGGAGGGGC |
|  | R: AGTGCAGGGTCCGAGGTATT |
| KLF16 | F: CGCCAAAGCCTACTACAAGT |
|  | R: CCTGCCAGTCACAAGCAAAAG |
| E-cadherin | F: GCTGGACCGAGAGAGTTTCC |
|  | R: CAAAATCCAAGCCCGTGGTG |
| N-cadherin | F: ATCCCTGCTTTCATTCTGACA |
|  | R: CAGTTGCTAAACTTCACTGAAAGG |
| β-actin | F: AGAGCTACGAGCTGCCTGAC |
|  | R: GGATGCCACAGGACTCCA5 |
| U6 | F: CTCGCTTCGGCAGCACA |
|  | R: AACGCTTCACGAATTTGCGT |
