# Supplementary material for: LncRNA LEF1-AS1 exerts a carcinogenic effect in breast cancer by accelerating proliferation, metastasis, and epithelial-mesenchymal transition
Source: Hereditas. 2025 Nov 29;163:7. doi: 10.1186/s41065-025-00613-2 (PMC12771952; doi:10.1186/s41065-025-00613-2)
Supplement: Supplementary file 2 — Supplementary Material 2. [file 41065_2025_613_MOESM2_ESM.docx]

1. The relationship between miR-328-5p and LEF1-AS1 according to regression analysis

|  | Model | β | P value |
| --- | --- | --- | --- |
| LEF1-AS1 | 1^a^ | -0.549 | 0.000 |
|  | 1^b^ | -0.559 | 0.000 |
|  | 1^c^ | -0.523 | 0.000 |

^a^: Unadjusted model; ^b^: Adjustment for age; ^c^: Adjustment for age, Tumor size, Lymph node metastasis, TNM stage.

2. The relationship between KLF16 and miR-328-5p according to regression analysis

|  | Model | β | P value |
| --- | --- | --- | --- |
| miR-328-5p | 1^a^ | -0.373 | 0.000 |
|  | 1^b^ | -0.374 | 0.000 |
|  | 1^c^ | -0.188 | 0.010 |

^a^: Unadjusted model; ^b^: Adjustment for age; ^c^: Adjustment for age, Tumor size, Lymph node metastasis, TNM stage, LEF1-AS1.

3. The relationship between KLF16 and LEF1-AS1 according to regression analysis

|  | Model | β | P value |
| --- | --- | --- | --- |
| LEF1-AS1 | 1^a^ | 0.464 | 0.000 |
|  | 1^b^ | 0.462 | 0.001 |
|  | 1^c^ | 0.380 | 0.001 |

^a^: Unadjusted model; ^b^: Adjustment for age; ^c^: Adjustment for age, Tumor size, Lymph node metastasis, TNM stage, miR-328-5p.
